# Sunscreens: A narrative review

**DOI:** 10.1002/ski2.432

**Published:** 2024-08-07

**Authors:** Hamisha Salih, Cristina Psomadakis, Susannah M. C. George

**Affiliations:** ^1^ University Hospitals Sussex NHS Foundation Trust Brighton UK

## Abstract

Sunscreens are topical formulations incorporating filters that protect our skin against ultraviolet radiation (UVR) emitted by the sun. Sunscreen use has been increasingly encouraged to protect against sunburn, skin cancer and photoaging that can occur because of prolonged and cumulative sun exposure. However, sunscreens and their constituent UVR filters have been purported to be problematic themselves. In this narrative review, we will describe the history of sunscreens, types of UVR filters and how sunscreens are classified and rated. We will also explore some of the controversies regarding sunscreens, including concerns about their safety and environmental impact. Awareness of these potential consequences is paramount to the process of informed decision‐making.

**What is already known about this topic?**

- Proper use of regular high‐factor sunscreen is recommended alongside other effective photoprotective measures to protect the skin against ultraviolet radiation (UVR) as prolonged exposure increases the risk of skin cancer and photoaging.
- Sunscreens are categorized as either organic or inorganic based on their UVR filter component. They are regulated differently across the world and have various rating systems for their effectiveness.

**What does this study add?**

- This narrative review explores the controversies regarding the use of sunscreens.
- Potential direct adverse effects of sunscreen use include hypersensitivity reactions, frontal fibrosing alopecia and hormonal disruption.
- Possible indirect effects of sunscreen use include impact on blood pressure and vitamin D deficiency.
- UVR filters have been found in almost all water sources, including treated water, posing a potential environmental threat.

## 1 INTRODUCTION

Sunlight exposes the skin to harmful ultraviolet radiation (UVR). Prolonged exposure can damage the skin and DNA, increasing the risk of skin cancers and premature ageing.^1^, ^2^, ^3^ To protect the skin against these consequences, sunscreens containing UVR filters are recommended alongside other photoprotection measures. Sunscreen products are commercially available to the public, and with a growing sunscreen market, it is important for clinicians and consumers to recognize the different components, rating systems and recommended application of sunscreens.

Certain UVR filters present in sunscreens, such as para‐aminobenzoic acid (PABA), can negatively impact health by causing hypersensitivity reactions.^4^, ^5^ There have also been controversies regarding the possible effects of systemic absorption of organic UVR filters,^6^, ^7^ impact on vitamin D production^8^ and association with frontal fibrosing alopecia (FFA).^9^ As part of our review, we will explore the potential health and environmental concerns associated with the use of sunscreens.

## 2 ULTRAVIOLET RADIATION

The sun emits optical radiation, which is energy within the electromagnetic spectrum comprised of three components: infrared, visible and ultraviolet. Their properties are determined by their wavelengths; the shorter the wavelength, the higher the energy output and the more damage they can cause.^10^ Optical radiation and its subdivisions are shown in Table 1.

**TABLE 1:** Optical radiation and its degree of penetration.^2^, ^11^

Percentage of sunlight	Radiation		Wavelength	Penetration
45%	Infrared	IR‐C	3000 nm–1 mm	To subcutis of skin
		IR‐B	1400–3000 nm	
		IR‐A	700–1400 nm	
50%	Visible	Red	625–700 nm	To dermis of skin
		Orange	590–625 nm	
		Yellow	565–590 nm	
		Green	500–565 nm	
		Blue	485–500 nm	
		Indigo	450–485 nm	
		Violet	380–450 nm	
5%	Ultraviolet	UVA	400–315 nm	To dermis of skin
		UVB	315–280 nm	To epidermis of skin
		UVC	280–100 nm	To stratosphere

UVR forms 5% of the sun's total radiation reaching the earth. Radiation is highest in summer and when the sun is highest in the sky, usually between 10 AM and 5 PM.^10^ It is greatest at the equator, decreasing in rising latitudes and highest at altitudes.^12^ UVR also penetrates water and reflects off surfaces such as snow. Environmental changes, such as air pollution leading to depletion in the ozone layer, also lead to increased radiation reaching the skin.^10^ The UV index is a measurement of UVR at a given time and location,^13^ and the effects of UVR exposure are dependent on intensity and duration.^11^

UVR can be divided into UVA (400–315 nm), UVB (315–280 nm) and UVC (280–100 nm) based on anthropogenic definitions.^10^, ^11^ With its shorter wavelength, UVC has the potential to cause the most damage. Fortunately, almost all UVC is absorbed by the ozone layer.^1^ UVA forms approximately 95% of the UVR penetrating the ozone to reach earth's surface and the remaining small proportion is UVB.^10^

UVB is responsible for sunburn that follows prolonged sunlight exposure, which is characterized by erythema.^1^ Exposure stimulates the generation of reactive oxygen species and damages cellular DNA directly, triggering mutagenesis and carcinogenesis. It also plays a role in vitamin D production in the skin and has immunosuppressive effects, hence the role of phototherapy in inflammatory dermatosis, such as psoriasis.^10^ Key skin chromophores, including DNA and amino acids, which absorb UVB are present mainly in the epidermis of the skin, therefore inhibiting UVB penetration beyond this layer.^14^

Most skin chromophores have poor UVA absorption abilities, allowing UVA penetration through to the dermis.^14^ UVA is filtered less by windows and clouds compared to UVB. UVA exposure predominately impacts the collagen fibres and elastin within the dermis, resulting in premature skin ageing which presents as atrophic skin, elastosis, deep wrinkles and telangiectasia.^3^ It also triggers melanogenesis, a protective mechanism to delay further DNA damage.^2^ Ultimately, the outcomes following prolonged exposure to UVA and UVB overlap, as both influence carcinogenesis as well as photoaging.^1^, ^10^

New areas of research have identified adverse effects of the other two components of optical radiation, visible light and infrared radiation. Evidence suggests that visible light contributes to hyperpigmentation, inducing darker and more persistent pigmentation in comparison to UVR, particularly in darker skin phototypes.^15^ Infrared radiation has also been reported to contribute to premature skin ageing.^3^

## 3 HISTORY OF SUNSCREENS

In some ancient cultures, the sun was considered a deity, worshiped as the source of energy. The ancient Egyptians were the first civilization noted to endeavour to photoprotect themselves with topical agents such as rice bran and jasmine.^16^, ^17^ With the application of mineral sunscreen, they sought to achieve a lighter skin tone which was associated with higher socio‐economic status.^17^ Other attempted methods of photoprotection have been described in ancient Greek and Native American civilizations, which include the use of olive oil and extracts from coniferous trees.^16^

UVR was discovered in 1801 by Johann Wilhelm Ritter and, over a century later in the 1920s, scientists Karl Eilham Hausser and Wilhelm Vahle demonstrated the tanning effects of UVA exposure and sunburn that results from prolonged UVB exposure.^17^, ^18^ This discovery led them to develop one of the first commercial sunscreens which comprised UVB filters; benzyl salicylate and benzyl cinnamate. Further sunscreens and filters emerged, including Eugene Shueller's Ambre Solaire by L’Oréal, which contained PABA.^16^, ^17^

In 1969, the photoaging effects of UVA were first described by the dermatologist Albert Kligman, who stressed the need for the development of UVA filters to be included in sunscreens.^19^ This was implemented a decade later, with avobenzone as the first UVA filter.^17^ Currently, the sunscreen market continues to expand with more sunscreen formulations available.

## 4 COMPONENTS OF A SUNSCREEN

A sunscreen is a product that protects the skin from radiation emitted by the sun.^20^ In Europe, these products are regulated by the European Commission, which regards them as cosmetic products.^21^ Whereas in the United States, products with photoprotective potential are regulated by the Food and Drug Administration (FDA) and are considered a drug. This means sunscreens are therefore subject to different regulatory and marketing requirements based on where they are retailed.^22^ The FDA categorizes sunscreen products as follows: category I, generally recognised as safe and effective (GRASE), category II, not GRASE, or category III, insufficient data to allow classification.^23^ This rigorous and timely process has led to fewer approved UVR filters in the United States (17) compared to Europe (29).^23^, ^24^, ^25^ The regulation of UVR filters differs across the globe, including within Asia, South America, Australasia and Africa, depending on whether they are classed as cosmetics or drugs.

Sunscreens are categorized as either inorganic or organic, depending on the photoprotective component in the formulation. Organic sunscreens contain filters that absorb UVR before the skin does and convert it to thermal energy, which is then released from the skin. A filter is considered organic if it is carbon‐based, and this does not equate to ‘natural’ nor do these filters decompose naturally in the environment. Inorganic sunscreens also protect skin by absorbing UVR, but they reflect visible light, which give them a more apparent appearance when applied. Table 2 lists the different UVR filters available.

**TABLE 2:** Ultraviolet radiation filters.^4^, ^23^, ^24^, ^25^, ^26^, ^27^

		Filter	INCI/other names	First available	Approved by	Main concerns
Organic	UVA	Meradimate	Menthyl anthranilate	1970	FDA	
		Oxybenzone	Benzophenone‐3	1978	EU, FDA. Banned in Hawaii	Photocarcinogenic, hormone disruption, free‐radical formation, absorbed by skin and breast milk detection
		Avobenzone	Butyl methoxy‐dibenzoyl‐methane	1988	EU, FDA	Photounstable, allergic contact dermatitis
		Ecamsule	Mexoryl XL, drometrizole trisiloxane	1997	EU	
			Mexoryl SX, terephthalylidene dicamphor sulfonic acid	2006	EU, NDA	
		Neo Heliopan AP	Bisdisulizole disodium, disodium phenyl dibenzimidazole tetrasulfonate	2000	EU	Allergic contact dermatitis
		Uvinul A Plus	Diethylamino hydroxybenzoyl hexyl benzoate	2005	EU	
		Mexoryl 400	Methoxypropylamino cyclohexenylidene ethoxyethylcyanoacetate	2020	EU	Absorbed by skin
		Piperazine	Bis‐(diethylaminohydroxybenzoyl benzoyl) piperazine	2021	EU	
	UVB	PABA	Para‐aminobenzoic acid	1949	Rated category II (not GRASE) by FDA	Allergic contact dermatitis, can increase the risk of cellular UVR damage and stains clothing
		Cinoxate	2‐Ethoxyethyl p‐methoxycinnamate	1961	FDA	Allergic contact dermatitis
		Octisalate	Ethylhexyl salicylate, octyl salicylate	1978	EU, FDA	Photounstable
		Homosalate	Homomethyl salicylate	1978	EU, FDA	Photounstable, absorbed by skin, hormonal disruption and breast milk detection
		Padimate O	Ethylhexyl dimethyl PABA, OD‐PABA	1988	EU, FDA	May indirectly cause DNA damage
		Uvinul N539	Octocrylene	1991	EU, FDA	Absorbed by skin, breast milk detection and free‐radical formation
		Octinoxate	Ethylhexylmethoxycinnamate, octyl methoxy‐cinnamate	1991	EU, FDA. Banned in Hawaii	Coral reef toxic, absorbed by skin, photounstable, hormonal disruption and breast milk detection
		Octyl triazone	Ethylhexyl triazone	1997	EU	
		Uvinul P25	Ethoxylated ethyl‐4‐aminobenzoate	1997	EU	
		Uvinul T150	Ethylhexyl triazone, octyl triazone	1997	EU	
		Amiloxate	Isoamyl p‐Methoxycinnamate	1997	EU	
		Enzacamene	4‐Methylbenzylidene Camphor	1998	EU	Oestrogenic effects
		Mexoryl SL	Benzylidene camphor sulfonic acid	1998	EU	Absorbed by skin
		Trolamine salicylate	Triethanolamine salicylate	1999	FDA category II (not GRASE)	Absorbed by skin
		Parsol SLX	Polysilicone‐15, dimethicone diethyl benzylmalonate	1999	EU	
		Ensulizole	Phenylbenzimidazole sulfonic acid	1999	EU, FDA	
		Sulisobenzone sodium	Benzophenone‐5	1999	EU	
		Mexoryl SO	Camphor benzalkonium methosulfate	2006	EU	Absorbed by skin
		Univul 400	Benzophenone‐1			Linked to breast, ovarian and prostate cancer, crosses blood‐placental barrier and hormonal disruption
	Broad‐spectrum	Sulisobenzone	Benzophenone‐4	1964	FDA	Absorbed by skin
		Dioxybenzone	Benzophenone‐8	1966	FDA	
		Iscotrizinol	Uvasorb HEB, diethylhexyl butamido triazone	1998	EU	
		Bisoctrizole	Tinosorb M, methylene bis‐benzotriazolyl tetramethylbutyl‐phenol	2000	EU	Photounstable
		Bemotrizinol	Tinosorb S, bis‐ethyl‐hexyloxyphenol methoxyphenyl triazine	2000	EU	
		Tinosorb A2B	Tris‐biphenyl triazine	2014	EU	
		TriAsorB	Phenylene bis‐diphenyltriazine	2019	EU	
Inorganic	UVB and shorter UVA wavelengths (320–340 nm)	Titanium dioxide	Ti02	1990	EU, FDA	White cast, can cause skin breakouts
	Broad‐spectrum	Zinc oxide	ZnO	1990	EU, FDA	White cast

Abbreviations: EU, European Union; FDA, Food and Drug Administration; GRASE, Generally Recognized as Safe and Effective; INCI, International Nomenclature Cosmetic Ingredient; NDA, FDA's New Drug Application; UVR, ultraviolet radiation.

The UVR filters in inorganic sunscreens include zinc oxide (ZnO) and titanium dioxide (TiO_2_). Their large particles form a visible white cast on the skin, forming a physical barrier. This feature enables inorganic sunscreens to act as effective UVR and visible light blocks; however, this often renders them cosmetically unacceptable.

Recently, formulations containing nanoparticles of ZnO or TiO_2_ have been developed. Micronizing the particles improves the cosmetic appearance but reduces its photoprotection ability against visible light.^28^ When micronized, inorganic sunscreens have a similar mechanism to organic sunscreens, that is, absorption of UVR.

## 5 SUN PROTECTION FACTOR

The increasing availability of sunscreens during the 20th century prompted chemist Rudolf Schulze to develop a method of rating their effectiveness in protecting against UVR damage.^17^ This was adapted by chemist and founder of Piz Buin, Franz Greiter, in 1978, who introduced the sun protection factor (SPF), which is used internationally today.^17^ The Australian Sunscreen Standard 2604 (AS 2604:1983) also pioneered and detailed the testing methods and procedures regarding the protective ability of sunscreen products.^29^

The global standard for in vivo testing of sunscreens is the International Organization for Standardization 24444:2019.^30^ SPF is established by applying 2 mg/cm^2^ of the sunscreen product on an area of the skin, typically the midback, and administering a series of five increasing doses of solar‐simulated radiation. The rating is then determined by dividing the dose of UVB that would cause sunburn with sunscreen by the dose of UVB that would cause sunburn without sunscreen. Therefore, in theory, applying a SPF 30‐rated sunscreen should mean that the received dose of UVB is reduced by 30 compared to no sunscreen being applied.

## 6 UVA RATING SYSTEMS

The UVA star rating system, developed in 1992 by Boots and Newcastle University, is a recognized system of determining sun protection against UVA.^17^ This is illustrated by the presence of one to five stars, symbolizing minimum to ultra‐protection, respectively.

An alternative symbol is UVA with an outer circle, which was established by The European Cosmetic and Perfumery Association (Colipa).^17^ It illustrates UVA protection in comparison to UVB in a product. A circle with UVA in the middle is permitted to be used on packaging if the UVA to UVB ratio is at least one third.^1^

Similar to SPF ratings for UVB, persistent pigment darkening is a comparable rating system used for UVA.^31^ It measures the ratio of minimal dose of UVA required to induce pigmentation in protected to non‐protected skin.^32^ However, it is important to consider that persistent pigmentation can be produced by mechanisms other than melanogenesis triggered by UVR, such as oxidative stress.

Additionally, the protection grade of UVA (PA) system, developed in Japan, utilizes plus symbols to indicate the level of UVA protection a sunscreen offers. This ranges from PA+ to PA++++, with the latter indicating the highest level of UVA protection.

## 7 SUNSCREEN APPLICATION

Sunscreen products come in several formulations including creams, lotions, sprays and sticks. They can also be incorporated into other cosmetic products, such as day cream, skin foundation and lip balm.

The SPF rating is based on the application of 2 mg/cm^2^ of the product.^33^ This is approximately seven teaspoons per application and 33 mL of product for full body application.^34^ However, studies have shown that the general population only applies an average of 0.5 mg/cm^2^, which means that the SPF on the label is not achieved.^33^, ^35^ Furthermore, sunscreens with lower SPF ratings demonstrate a linear dose–response relationship, whereas with high SPF sunscreens, photoprotection decreases almost exponentially as the applied quantity decreases.^36^ Sunscreens should therefore be applied generously to the skin and without gaps to ensure uniform coverage.^37^ Most frequently missed areas include the neck, ears and temples.^35^ SPF determined using UVR solar simulators have also been shown to overestimate the SPF ability of sunscreens when used in natural sunlight, potentially due to a mismatch of spectral emissions.^38^

Sunscreen should initially be applied 15–30 min before sun exposure and then reapplied 15–30 min after sun exposure has started, to increase the amount of the product on the skin. Two hourly reapplications are recommended and should be more frequent in the presence of vigorous activity or reapplied after swimming.^33^, ^37^ In Europe and Australia, an expiration date is not a requirement on product labelling, but sunscreens should ideally be used within 3 years unless stated.^32^

National Health Service in the United Kingdom advises applying a sunscreen with SPF 30 and UVA 4‐star protection rating or above, particularly during the months of March to October when the UV index is likely to be higher.^39^ The pillars of sun protection include avoidance of sunlight, seeking shade and covering skin with clothing. Sunscreens should be used as an additional measure in combination with these, rather than a solitary measure, and should not be used to extend the duration of sun exposure.

## 8 SUN PROTECTIVE CLOTHING

Hats, particularly those with large brims, are recommended to protect the head and neck from UVR.^13^ Covering the skin with clothing provides protection by absorbing and reflecting radiation from the sun. The level of protection is rated using the ultraviolet protective factor (UPF), which assesses the amount of UVR able to penetrate dry and non‐stretched clothing.^3^ UPF is the factor by which UVR exposure is reduced by, starting from 15. Regular clothing can provide a level of protection based on the nature of the fabric, with denser materials providing more UVR protection. Specially designed sun protective clothing may have properties devised for wear in warm conditions such as moisture wicking and ventilated weaves. In the United States, an item of clothing labelled as protective against UVR must be thoroughly tested in conditions that mimic the effect of repetitive use on UVR protection. This includes laundering, which reduces the density of the fabric, and exposure to chlorinated water.^40^

## 9 POTENTIAL DIRECT ADVERSE EFFECTS OF SUNSCREENS

### 9.1 Contact dermatitis

Contact dermatitis that results from sunscreen use can be due to contact allergy or due to a photoallergic reaction. A photoallergic contact dermatitis reaction occurs in previously sensitized patients following UVR or visible light exposure on an area of skin with topical photoallergen applied. An antigen is generated when the exogenous compound from the sunscreen is combined with a protein within the skin in the presence of sunlight, which eventually leads to activated T‐lymphocyte cells in the skin.^5^ Diagnosis of this is achieved following photopatch testing using a sunscreen series, which tests the photo allergens present in sunscreens.^5^

In 1956, issues with UVR filters were first reported, with patients increasingly presenting with delayed hypersensitivity contact allergies to PABA.^17^ This reaction to PABA was due to the breakdown oxidation products within the epidermis.^41^

Aside from UVR filters, sunscreens contain substances such as emollients, preservatives, emulsifiers and fragrances. Organic sunscreens, in particular, contain ingredients to improve application and cosmetic appearance of the product on the skin, with potential for allergic or irritant dermatitis.^5^ The most common cause of allergic or irritant dermatitis is oxybenzone (benzophenone‐3).^4^ Other culprits include PABA, cinoxate, avobenzone and other forms of benzophenones.

Whilst contact dermatitis reactions secondary to UVR filters are a concern, evidence suggests that their incidence is low.^42^

### 9.2 Effects on endocrine activity

UVR filters have been detected in wastewater as well as surface water.^43^ Organic UVR filters have also been found in fish, indicating that these chemicals are entering the food chain. This has raised concerns associated with biomagnification and bioaccumulation of UVR filters and the potential impact on human health, such as developmental and systemic changes.^44^ Studies have also demonstrated systemic absorption of topical sunscreen ingredients, which triggered a guideline review by the FDA and the requirement for further systemic safety testing.^7^, ^45^, ^46^

Additionally, animal studies have shown an effect on endocrine activity in fish and development in rats and fish following exposure.^47^ Specifically, disruptions in oestrogen, androgen and thyroid hormones have been identified in vitro and in vivo studies.^44^, ^48^ Transdermal passage of UVR filters, particularly chemical‐based ingredients, have also been found in human breast tissue and breast milk as well.^43^ Oxybenzone, for example, can be absorbed by the skin and into the blood, crossing blood–brain and blood–placental barriers.^6^, ^48^ However, there is minimal penetration of nanoparticles used as mineral filters beyond the stratum corneum layer of the skin.^49^

Potential adverse health effects of the systemic presence of these chemicals remains uncertain. However, the likelihood of this causing harm is considered negligible and evidence does not suggest that individuals should avoid the use of sunscreens.^7^, ^50^

### 9.3 Frontal fibrosing alopecia

Organic sunscreens have recently been investigated for their potential role in the pathogenesis of FFA, first described in 1994 as progressive, permanent hair loss affecting the frontal and temporal areas.^51^ As a newly defined entity with a rising prevalence, multiple hypothesis for its pathogenesis have been suggested. A possible explanation may be the increased use of unidentified topical triggers in sunscreens and cosmetics. A meta‐analysis exploring the correlation of sunscreen use with the incidence of FFA concluded that sunscreen users are 2.21 times more likely to develop FFA.^9^ However, this association remains controversial and there is insufficient evidence to describe the relationship as causal.^52^

## 10 POTENTIAL INDIRECT ADVERSE EFFECTS OF SUNSCREENS

### 10.1 Vitamin D deficiency

Skin exposure to UVB is vital for vitamin D production. Synthesis occurs in the epidermis following exposure and is required for good general health, including musculoskeletal health and reduced risk of malignancies.^8^

Laboratory studies with artificial UVR, with distributing spectrums between 260 and 360 nm, support the theorized risk of sunscreen causing vitamin D deficiency.^53^ However, evidence from observational studies and randomized trials suggest that the risk of vitamin D deficiency is low and synthesis is maintained with sunscreen use in real world environments and natural sunlight.^53^ Other studies have concluded that regular sunscreen use is considered to not compromise vitamin D levels in the population. However, individuals with rigorous photoprotection behaviours are at risk of lower levels of vitamin D.^54^

Regular use of high‐factor sunscreen use and its effect on the concentration of vitamin D is yet to be investigated in a high‐quality clinical trial.^53^ For patients with darker skin phototypes at higher risk of vitamin D deficiency, there are no data which evaluate this risk against skin cancer.^53^ Until this information is available, uncertainty regarding sunscreen use will persist and therefore undermine its benefits, despite the majority of evidence demonstrating only theoretical concerns.

### 10.2 Blood pressure

Seasonal variation in blood pressure (BP) occurs, with systolic and diastolic BP lower in summer than winter. Distance from the equator also impacts the mean population BP, with those at higher latitudes having higher BP. Additionally, it has been shown that sun exposure can contribute to lowering BP. This may be due to the export of nitric oxide stored in the skin into circulation following UVR exposure, leading to a decrease in circulating nitrate and a rise in nitrite concentrations. Another causative mechanism could be the vasodilation effects following sunlight exposure.^55^ These findings showing reduced BP and lower rates of cardiovascular disease in individuals with higher UVR exposure suggest that these haemodynamic benefits should be considered in the risk versus benefit assessment of sun exposure. The possibility of whether insufficient exposure to UVR as a potential risk factor for hypertension should be considered^56^ and, as sunscreen application is aimed at reducing UVR exposure, further research evaluating regular high‐factor sunscreen use and its impact on BP is required.

### 10.3 Increasing skin cancers

Despite the increasing availability of sunscreens and photoprotection, skin cancers are rising in incidence, including cutaneous malignant melanomas (CMM), which have increased by over 40% since 1990s.^57^ The aetiology of CMM is multifactorial and include modifiable factors such as UVR exposure.^58^ Consumers may select sunscreens based on their SPF rating. However, inadequate application may exponentially decrease the level of photoprotection. Studies have shown that people may prolong their sun exposure after applying higher SPF sunscreen, erroneously believing that they are sufficiently protected, and therefore increasing the risk of skin cancers.^59^

Individuals with known skin cancers may also begin utilizing sunscreen following diagnosis, and therefore the interpretation of rising CMM can be difficult given the lack of cause and effect. The lag time between UVR exposure and development of CMM also complicates this interpretation, given probable variability in sunscreen use over time.

Additionally, the published evidence exploring the association between sunscreen use and melanoma genesis is weak.^60^ Many epidemiological trials have not been able to adequately evaluate the effectiveness of sunscreen use on CMM incidence, and sunscreen use is mostly justified by evidence relating to UVR exposure effects on the skin.^61^

Furthermore, whilst regular high‐factor sunscreen use is recommended for all skin types to reduce the risk of skin cancers, further research is required to inform on the effectiveness of this in skin of colour, given the lack of evidence for prevention in this group.^62^

## 11 ENVIRONMENTAL CONCERNS

Plant‐derived compounds with aromatic rings have been used in sunscreen products.^63^ These compounds can absorb UVR as well as exhibit antioxidant properties.^63^ However, the classification of sunscreens based on their carbon content as either organic versus inorganic should not be misinterpreted as ‘natural’ or environmentally conscious.

Increasing sunscreen use is thought to impact our environment as UVR filters have been found in almost all water sources due to difficulties removing components using standard water cycling processes.^64^ The environmental impact of some UVR filters, such as octocrylene and butyl methoxy‐dibenzoyl‐methane (avobenzone), has been reviewed in detail.^65^ However, further studies exploring the long‐term effects of UVR filters in the aquatic environment are needed.

It is also thought that some UVR filters, such as oxybenzone and octinoxate, play a role in coral reef bleaching and threaten the aquatic ecosystem, which has led to their ban in certain countries.^64^, ^66^

However, there is limited evidence demonstrating a direct impact of sunscreen causing coral bleaching in the real world.^67^ Other factors, such as global warming and decreasing ocean salinity, are thought to be major contributors to coral reef bleaching.^64^ Further research is needed to evaluate the risk to corals posed by UVR filters.

## 12 CONCLUSION

There are clear health implications that follow prolonged sunlight exposure and photoprotective measures, including sunscreen application, are required to reduce skin cancer risk and photoaging. Clinicians and patients must be aware of different types of sunscreens and their recommended application to achieve optimal benefits from use.

Exposure to UVR can be damaging; however, adverse effects due to sunscreen use has also been purported. Many UVR filters are relatively new, with their long‐term health and environmental effects unknown. Open communication between manufactures, cosmetic companies, environmentalists, regulatory bodies and dermatologists is vital to ensure optimum patient and environmental outcomes.

## CONFLICT OF INTEREST STATEMENT

Dr George reports paid consultancies with Almirall and Leo Pharma, receiving speaker fee from Pfizer and L’Oréal, and receiving sponsorships to attend meetings from Sanofi and Almirall. Dr Psomadakis has had commercial contracts with L’Oréal and Aveeno. No other conflicts of interests have been declared.

## AUTHOR CONTRIBUTIONS

**Hamisha Salih**: Writing—original draft (lead). **Cristina Psomadakis**: Supervision (supporting); writing—review and editing (equal). **Susannah M. C. George**: Conceptualization (lead); supervision (lead); writing—review and editing (equal).

## ETHICS STATEMENT

Exempt from the IRB approval as this review article did not involve human or animal subjects.

## PATIENT CONSENT

Not applicable.

## Data Availability

Data sharing is not applicable to this article as no new data were created or analysed in this study.
